# Causing trouble and being transmissible: COVID-19 survivors’ experiences of stigma and discrimination in South Korea

**DOI:** 10.3389/fpsyt.2023.1103572

**Published:** 2023-03-14

**Authors:** Jiyeon Kang, Hyang Soo Kim, Hyun Ji Yi, Yesung Lee, So Hee Lee, Kyoung-Ho Song, Hye Yeon Park, Hong Sang Oh, Doran Yoon, Pyoeng Gyun Choe, Eun Joo Lee, Chi-Hyun Choi, Minyoung Sim, Eun-Seung Yu, Jong-Woo Paik, Hye Yoon Park

**Affiliations:** ^1^Department of Anthropology, Seoul National University, Seoul, Republic of Korea; ^2^Department of Nursing Science, Sungshin Women’s University, Seoul, Republic of Korea; ^3^Department of Psychiatry, Seoul National University Hospital, Seoul National University College of Medicine, Seoul, Republic of Korea; ^4^Department of Psychiatry, National Medical Center, Seoul, Republic of Korea; ^5^Division of Infectious Diseases, Department of Internal Medicine, Seoul National University Bundang Hospital, Seoul National University College of Medicine, Seongnam, Republic of Korea; ^6^Department of Psychiatry, Seoul National University Bundang Hospital, Seoul National University College of Medicine, Seongnam, Republic of Korea; ^7^Division of Infectious Diseases, Department of Internal Medicine, Armed Forces Capital Hospital, Seongnam, Republic of Korea; ^8^Division of Infectious Diseases, Department of Internal Medicine, Seoul National University Hospital, Seoul National University College of Medicine, Seoul, Republic of Korea; ^9^Department of Pediatrics, Yonsei University College of Medicine, Seoul, Republic of Korea; ^10^Department of Psychiatry, SMG-SNU Boramae Medical Center, Seoul, Republic of Korea; ^11^National Center for Disaster and Trauma, National Center for Mental Health, Seoul, Republic of Korea; ^12^Department of Counseling Psychology, The Cyber University of Korea, Seoul, Republic of Korea; ^13^Department of Psychiatry, Kyung Hee University Hospital, Kyung Hee University College of Medicine, Seoul, Republic of Korea

**Keywords:** COVID-19, stigma, South Korea, psychosocial distress, emerging infectious disease, pandemic

## Abstract

**Background:**

The stigma associated with coronavirus disease (COVID-19) is relatively neglected in policies for handling the disease. Stigmatization occurs only within specific social contexts in local societies.

**Objective:**

This study aims to examine COVID-19 survivors’ experiences of social stigma and discrimination in South Korea in the first 2 years of the pandemic.

**Methods:**

Semi-structured interviews were conducted.

**Results:**

Of 52 participants, 45 reported that they had to cope with stigma and discrimination in their intimate social relationships, workplaces, and children’s schools, ranging from subtle actions to job loss. Sexual minorities who were involved in mass disease transmission in the early part of the pandemic experienced a higher level of stigmatization. The stigmatization dealt with in this study was related to two themes: survivors’ sense of causing trouble and possibility of transmission.

**Conclusion:**

By intertwining this stigma with the experiences of public health measures through the voices of survivors, this study reveals the local context of East Asia in terms of culture-specific aspects of COVID-19-related stigma.

## 1. Introduction

Emerging infectious diseases (EIDs) are accompanied by stigma (1, 2), and coronavirus disease (COVID-19) is no exception. Since January 30, 2020, when the World Health Organization (WHO) declared this outbreak to be an international public health emergency, governments around the world have exercised all their authority to curb the spread of the virus. While controlling this infectious disease has become the top priority in political, social, economic, and public health sectors, the stigma associated with COVID-19, which can be seen throughout the world, is relatively neglected in policies for handling this disease (3).

Stigma is defined as an defining characteristic of disgrace that is related to a particular context, quality, or person. It is a deleterious label that makes the stigmatized person or group feel secluded from main stream society and that can further lead to segregation, devaluation, and discrimination (4–8). Since the Erving Goffman’s seminal work (5), stigmatization has been explored in various area, ranging from poverty, mental disease, and disability to gift in childhood. Stigma can be classified into three types: enacted stigma, perceived stigma, and internalized stigma (8). Enacted stigma refers to actual negative actions experienced by the stigmatized person or group, whereas perceived stigma means the anticipation of being discriminated against. If the stigmatized person or group accept a negative stereotype and act in a way that endorses this stereotype, this refers to internalized stigma. To avoid the stigmatization, the stigmatized person or group may adopt diverse strategies, such as passing as normal, shunning the stigmatized, and selective disclosure (5).

Disease-related stigma is originated fear of infection and death, disfiguring caused by the disease and ignorance of the cause, infectivity and the nature of the disease (9). Particularly, as an emerging respiratory infectious disease, COVID-19 evokes the fear of infection and dying that is associated with ignorance of the disease. Although in some cases people can positively appropriate stigma as an identity and self-esteem as defined “stigma allure (10),” stigmatization in general has the power to harm certain groups’ or individuals’ psychological and social wellbeing. The stigma and discrimination related to diseases impact survivors’ mental health, and the infected may even hide their symptoms or fail to cooperate in getting treatment to avoid stigmatization (11, 12). Due to its adverse effects, stigma is even considered an independent social determinant of health (7, 13). Most of all, stigma impacts not only the stigmatized themselves but also the caregivers, families and friends, and the whole society (14). In terms of stigma, patients and survivors are able to conceal their infectious history while the preventive measures endangers them to be disclosed. COVID-19-related stigma can be classified into personal traits one and situational one.

As seen in the cases of HIV (15, 16), Ebola virus (17, 18), and SARS (19), survivors of emerging infectious diseases have faced social exclusion and blame (20) and discrimination against privileges and opportunities (9), which led to the affected patients and healthcare workers’ psychological distress and post-traumatic stress (8, 21, 22). Similarly, since the onset of the COVID-19 pandemic, verbal and physical attacks and avoidance have been perpetrated against individuals of Chinese descent, other Asians, healthcare workers, COVID-19 patients and survivors, and the family members of patients in Latin American countries, European countries, the U.S., India, and some African countries (1, 2, 23–26). When it comes to public discourse scapegoating foreigners and travelers—individuals who are commonly singled out for their responsibility in the spread of the virus—COVID-19-related stigma can be found everywhere (27).

Nowadays, no one in the public health sector would disagree that disease-related stigma is as harmful as the disease itself and that we should be prepared for effective stigma-reduction interventions for the future occurrence of EID. To do so, it is crucial to understand what attributes were attached to COVID-19 survivors and how stigmatization was enacted in local societies. Stigma is recently explored as situational social process. In other words, although stigmatization can be observed everywhere and at any time (28) and the biological traits of disease may be universal, individual stigmatization occurs only within specific social contexts in local societies (12, 29, 30). In the case of COVID-19 pandemic, preventative measures, ethnicity dynamics, the medical capacity, and other cultural beliefs and political stances may impact the ways in which stigmatization enactivated in each society in different ways. For example, in Euro-American countries, the public fear of COVID-19 cast Asians as “others” (31–33), and cultural ideas and norms regarding wearing masks facilitate avoidance behavior and even physical attacks on those wearing masks in public spaces. By contrast, in East Asian countries, the social stigma regarding COVID-19 has unfolded in different ways. For instance, in South Korea and China, where people are used to wearing masks to prevent infectious diseases and protect themselves from inhaling fine dust, those who do not wear masks are declined access to public spaces. The degree and type of preventive measures that each government has adopted have also affected how laypeople make sense of the pandemic (34).

South Korea is well-known for its rapid and nation-wide preventative interventions. Wearing masks both indoor and outdoor was mandatory and people cooperated with the government-leading policies while the fatality of COVID-19 remained low. This paper examines how COVID-19 survivors in South Korea experienced social stigma and discrimination in the first 2 years of the pandemic. By probing what COVID-19 survivors went through from the time of infection to after recovery, this paper answers the following questions: (1) What kind of stigmatization, either perceived or enacted one, did survivors encounter? (2) What was the attributes of stigma that were attached to COVID-19 survivors in South Korea? (3) In what social relationships and under what circumstances did survivors experience stigmatization? By seeking answers to those questions, we would like to make suggestions to prevent future EID-related stigma.

## 2. Materials and methods

### 2.1. Design

This study was part of a large-scale research project exploring COVID-19 survivors’ experiences and psychosocial distress related to COVID-19 treatment, quarantine/isolation, and post-recovery experiences. Semi-structured, in-depth interviews were conducted with the participants.

### 2.2. Recruitment

Coronavirus disease survivors were recruited from six hospitals in Seoul and Gyeonggi-do. A total of 61 participants were recruited through their attending physician’s suggestions, advertisement flyers distributed to the hospitals, and snowball sampling. Among the recruited patients, 52 participants finally enrolled and participated in this study; nine patients lost contact for unknown reasons. All participants were informed about the purpose and methods of the study. Written consent was waived by the hospitals’ IRB because the study took place when the survivors were reluctant to meet in person due to their deteriorated condition, fear of stigma, and the possibility of a second infection. Instead, verbal consent was recorded at the beginning of each interview. All interviews except one were conducted *via* phone.

In the research preparation stage, a list of questions was prepared to guide the semi-structured interviews (Table 1). The interviews were designed to follow the narrative flows of participants from the appearance of symptoms to their return to everyday life. The themes of stigma appeared through natural narrative flows, in particular, in the questions regarding concerns, disclosure, and changes.

**TABLE 1:** The interview questions.

General experiences of COVID-19 infection
“I would like to understand your experience of COVID-19 since the appearance of symptoms to post-discharge.”
**Responses from others**
“Who knows about your infection and how did they react to the news?”
“What did you feel about the reaction?”
**Disclosure**
“Did you voluntarily disclose your infection to people? If yes, to whom did you disclose it?”
“Was there any involuntary disclosure? If yes, what situation led to the disclosure?”
**Concerns and suffering**
“What was your concern while going through the infection?”
**Support**
“Who (or what) helped and supported you?”
**Changes**
“Have you experienced any changes since your infection?”
**Suggestion**
“What could have helped you return to everyday life after the COVID-19 infection?”

Four members of the research team, with experience in qualitative research in the fields of medical anthropology, gender studies, and pedagogy, conducted the semi-structured interviews. With the participants’ approval, the interviews were recorded and transcribed. All identifying data were deleted from the transcripts. Theoretical saturation was reached when no new issues were addressed in the interviews, and consequently, recruitment was closed.

### 2.3. Analysis

The transcripts were analyzed based on grounded theory (35). In the open coding stage, four members of the research team independently created core categories and key themes using memos that emerged while iteratively reading the transcripts. Through constant discussions, these categories and themes were integrated into one chart, and a detailed guideline for coding was shared with other team members. According to the integrated codes and themes, the entire interview transcription was cross-reviewed by the team members, and the codes and themes were revised again through discussions. The final comprehensive code structure of the relationships between the core categories and themes was approved by all research team members. Through discussions, reviews, and supervision, all team members, having diverse qualifications (M.D., Ph.D., doctoral candidate, and B.A.) and backgrounds (psychiatry, medical anthropology, gender studies, and pedagogy), examined the interview data in a reflexive manner; this process contributed to minimizing biased interpretations.

### 2.4. Ethics

The ethics committees of Seoul National University Hospital (No. H-2010-048-1163), Seoul National University Bundang Hospital, National Medical Center, Armed Forces Capital Hospital, Seongnam Citizens Medical Center, and Boramae Medical Center approved the study protocol and materials between April and June 2020. This study adhered to the Declaration of Helsinki. All names used in this paper are pseudonyms.

## 3. Results

### 3.1. Demographic features

In total, 52 COVID-19 survivors participated in the study between February 2020 and October 2021. All interviews lasted 60–90 min. The participants’ demographic characteristics are displayed in Table 2.

**TABLE 2:** Demographic features.

		*N = 52*	
		***N***	**%**
Gender	Male	23	44.2%
	Female	29	54.8%
Age	≤19	1	1.9%
	20–29	5	9.6%
	30–39	9	17.3%
	40–49	10	19.2%
	50–59	9	17.3%
	≥60	18	34.6%
Working/Student	Working	36	69.2%
	Student	1	1.9%
	N/A	15	28.8%
Working status	Self-employed	15	28.8%
	Employed	20	38.5%
	Others	1	1.9%
Date of infection	Jan to Dec, 2020	20	38.%
	Jan to Mar, 2021	23	44.2%
	Apr to Aug, 2021	9	17.3%
Hospitalization period	≤14 days	24	46.2%
	15–30 days	20	38.5%
	≥30 days	8	15.4%
Psychiatric intervention during hospitalization		5	9.6%
Cessation of work		15	28.8%
Move		0	0%
Acquaintance’s death		0	0%
Public disclosure of infection		14	26.9%

### 3.2. Experiences of COVID-19 test and treatment process

The period in which the participants were infected span between January 2020 and August 2021 was the time that “3T policy” and social distancing policy of the Korea Disease Control and Prevention Agency (KDCA) were strongly enforced until the third vaccination (2021.10.12) and deregulation of social distancing (2021.11.1) began (36). The 3T policy, which was adopted by KDCA in the first 2 years of the COVID-19 pandemic, entailed preemptive testing, prompt tracing, and proper treatment. Along with test, trace, and treatment, isolation of patient and quarantine of those who had physical contact with the patient were strictly enforced. The details of this policy that impacted the participants are outlined in the following.

The participants took a PCR test when symptoms such as coughing, fever, and muscle pain appeared or when they had physical contact with a confirmed COVID-19 patient. After receiving PCR tests at screening centers, which were installed at hospitals, public health centers, schools, and sports facilities, near their homes or workplaces, they received positive results the same day or at least the next day. Subsequently, they were soon transferred to a hospital isolation ward or residential treatment center *via* ambulance; the latter was reserved for those with only minor symptoms.

After the participants were taken away, their homes were disinfected by the public health center staff. In the treatment center or hospital, all physical contact with the outside world was prohibited, except for extremely limited contact with medical staff. Voice/video calls, the internet, and TV were allowed. If the participants no longer had severe symptoms 10 days after being admitted, they were discharged.

Once a COVID-19 patient was identified *via* PCR test, everyone who had physical contact with the patient in the last 3–7 days also received a PCR test. Those who tested positive were taken to a hospital or residential treatment center, where they had to remain for at least 10 days. Those who were in close contact with a patient also had to self-quarantine at home for the incubation period (14 days), even if they had a negative result. If a negative result was obtained on the last day of quarantine, the at-home quarantine order was lifted. Some patients and individuals in isolation were provided with short-term emergency COVID-19 relief funds for their labor loss.

An epidemiological investigation was conducted after each positive result. Through phone calls, epidemic intelligence officers (epidemiological investigators) asked the participants about their whereabouts, whom they had met, and whether they had followed the COVID-19 guidelines during their incubation period. In addition to the interviews, the epidemiological investigators sometimes reviewed CCTV footage and patients’ credit card records. Those who were identified as close contacts of infected patients were tested for COVID-19.

From the onset of the COVID-19 pandemic, the KDCA disclosed statistics (e.g., the number of confirmed cases, the rate of severe cases, the number of recoveries, and the fatality rate) in press briefings and on its website every day. Each patient was given a case number by the local government. The local government sent a text message to all residents every morning to notify them of the number of new and accumulated confirmed cases in the local district. The recent routes of each case number were disclosed on the KDCA and local government websites. In the early phase of the pandemic, each patient’s routes and times of visitation at each location were disclosed; however, as concerns about privacy infringement increased, information about newly infected patients’ routes was collectively disclosed so that an individual patient’s route was untraceable.

The requirement to wear a mask in public spaces, including in public transport vehicles, movie theaters, religious facilities, educational institutes, schools, bus, and train stations, and department stores, was enforced beginning in May 2020. Enforcement was limited to indoor places, but people always wore masks both indoors and outdoors. Public facilities, such as restaurants, provided thermometer machines and hand sanitizers at their entrances. All visitors were supposed to fill in entry logs (or tag a QR code) to access the facilities, and these logs were used to trace close contacts whenever a patient was confirmed.

### 3.3. Experiences of stigma and discrimination

#### 3.3.1. Common features

Of the 52 participants, 45 (86.5%) reported that they recognized perceived stigma against COVID-19 survivors and that this expectation was a major concer after being infected. From the moment of diagnosis, they internalized COVID-19-related stigma. They felt as if they became “conspicuous” and “separated from ordinary people,” and they were afraid that people would see them as “virus carriers.” Mr. Eom (#031), in his early thirties, remarked, “I feel like no one will try to set me up on dates anymore because I caught COVID-19.” Feelings of guilt, shame, and labeling were experienced, as Ms. Hahm (#058), a woman in her forties and a senior staff member at her workplace, described: “When I went back to my workplace, even though no one mentioned my infection, I felt as if a scarlet letter was on my chest. At lunchtime, I could not hold my head up in the cafeteria. I felt guilty. The effect of self-stigmatizing was powerful.”

Of those 45 participants, 37 reported experiences of enacted stigma as well. The scope and type of enacted stigma addressed by the participants included both subtle and indirect reactions, such as uncomfortable looks, rumors, and attempts to uncover the infected, and obvious and direct reactions, such as avoidance, termination of a relationship, expressions of fear against the survivors, public blame, and job loss. As consequences of stigma and discrimination, the participants reported depression, anxiety, feeling of isolation, guilt feeling, psychological shrinking, and suicidal thought. Some of the participants reported that the perceived stigma delayed them to get a test promptly.

The attributes of COVID-19-related stigma appeared as two themes: the sense of causing trouble and the possibility of transmission. First, the participants shared the idea that COVID-19 patients ended up creating “big troubles” for people around them by causing others to get tested and quaranteened, become infected, and be hospitalized. The participants tended to internalize this attribute and consider themselves, in part, contributors to others’ “troubles,” and some of them were faced with overt blame for their “carelessness.” For instance, Ms. Ahn’s (#029) neighbor accusingly commented, “You could have avoided catching COVID-19 if you wore a mask properly and washed your hands. Didn’t you wear a mask?” With the sense of responsibility the participants felt for their infection and transmission of the virus, it was critical to their psychological distress whether they actually caused an additional infection. Mr. Koo (#012) expressed relief that he did not cause any additional infections, saying, “I was much relieved that no one was infected by me. That was why I could go back to everyday life quickly.” In contrast, Mr. Jang (#009), in his seventies, blamed himself, “Because of me, five family members out of six were infected. It was beyond expression.”

In the participants’ accounts, COVID-19 survivors were classified into two groups: innocent victims and individuals responsible for their own infection and after management. For example, if a patient became infected despite their best efforts to follow preventive policies (e.g., by refraining from eating out and wearing a mask in all circumstances) and tried to minimize the scope of close contact with people (e.g., by preemptively getting tested), they deserved to receive enough sympathy and support. However, if a patient visited multiple places while having symptoms or concealed some locations they had visited during the epidemiological investigation, they deserved criticism. While interacting with people in their everyday lives, such as in conversations with neighbors and through online forums, the participants learned about this classification and internalized it both before and after their infections. Ms. Joo (#056) recalled how she recognized people blamed for COVID-19 patients before her own infection, “I kept reading things on the mom forum, like critical comments, and it was like a witch hunt. I was very nervous. People on the forum blamed those who did not follow the rules, such as those who wandered around after their test.”

By going through the screening, treatment, and quarantine/isolation, the participants learned that it was impossible to prevent infection and transmission through perfect self-discipline and that rules may often be violated without selfish intentions. Yet, they reflected with anxiety on whether their “carelessness” caused the infection. Simultaneously, the participants tried to differentiate themselves from the “irresponsible” survivors, thinking that it would be unfair if they were generalized as “the careless infected.” For instance, Mr. Choi (#004), who was infected by an unknown source, said, “It would be less unfair if I went to a pub, but I did not. I felt it was very unfair.” In constrast, Ms. Min (#019) felt guilty and blamed herself. She was infected by a taxi driver and passed it on to her entire family. She believed that the adversity of her family resulted from her negligence of hygiene. “My friend and I took the taxi and had a lot of conversation with the driver. I did not wash my hands right after going home. I think that was the problem because my friend was not infected.”

This attribute of causing trouble could be applied to those who passed COVID-19 on to the participants. Although many participants commented on those infected them, “They did not do it intentionally,” some participants criticized their carelessness. Mr. Paeng (#056) did not hide his anger against his colleague at work. Four of colleague went on a trip together during a weekend and were infected. Even though they all had a fever, they took a Tylenol and went to the office, which led a dozen of infection cases at the company. “I was so pissed off and was going to sue them. I wanted to kill them in the ICU. I still feel anger. When I hear their voice at work, I feel rage.”

The second attribute of COVID-19-related stigma was a collective fear that recovered patients might still be able to transmit the virus because some “dead virus” could remain in their bodies. Not to mention the people around them, the participants were also unsure whether they were “totally safe” to others. Hence, many participants commented that they understood their friends’ and neighbors’ avoidance and voluntarily tried to avoid physical contact for a while. Ms. Moon (#018) reported, “Because we had been infected, I was hesitant to meet people. We (she and her children) could not go to the playground, of course, and when we encountered people there, I kept a distance and told them, “I was isolated. Would it be okay? You’d better keep away from me.” I did this for a couple of weeks.”

The discharge process also reinforced the fears of transmissibility, as the participants did not receive any tests when they were released. Instead, they were informed by the medical staff that they would test positive for a while because of the remaining virus in their bodies. This explanation of the remaining virus and the process of discharge without assurance failed to end the anxiety and fear of the participants and the people around them. Mr. Hong (#005), a man in his sixties, said, “People asked, ‘Did you receive the negative result or not? Can’t we trust my safety only after you test negative?”’

This no-test discharge policy caused problems, particularly in schools and workplaces. Due to the fear of transmission, some schools and workplaces required the participants to submit a negative result, and the KDCA and the healthcare center did not endorse the participants’ return in an official way. Some participants, thus, had to wait an additional couple of weeks for their negative results, which jeopardized their job stability. We will return to this issue in “section 3.3.3 Contexts in workplaces.”

#### 3.3.2. Contexts within private networks

The participants’ infections were known to their relatives, friends, acquaintances, and neighbors through voluntary disclosure or involuntary disclosure such as official notice, transportation and disinfection processes, rumors, and notices from educational institutes (e.g., kindergartens). This category was most frequently addressed as the stigmatizers. Compared to their workplaces, their private social networks were places where the participants could control information disclosure to some degree. They carefully selected to whom and in what circumstances they disclosed this information and often chose to hide their infection to avoid uncomfortable situations. The scope of disclosure varied, ranging from no one (including family members) to only family members and close friends or to those the participants interacted with in daily life. Some participants thought that not telling about the infection could be considered a lie and they were reluctant to meet people for a while to avoid this distress.

Because of the intimate nature of their relationships, the participants’ relatives and friends tended to explicitly reveal their fear of COVID-19 and blame the participants for “the trouble.” For example, Ms. Yim (#036), a woman in her thirties, was infected by an unknown source. She had met many relatives and friends before her positive COVID-19 result since it was during a holiday break. Among the friends she had met, only one was infected, and the rest tested negative. However, her friends still suffered economic damage due to quarantine and isolation. Ms. Yim’s friend’s husband called Ms. Yim and swore at her. The rest of Ms. Yim’s friends did not blame her to her face, but she heard from others about the difficulties her friends endured. When she gathered up the courage to call some of her friends, she sensed that they were avoiding her. Ms. Yim felt very regretful that she could no longer reach out to her friends.

Ms. Yim’s parents-in-law were also forced to be quarantined at home. This 2 weeks quarantine caused such great psychological distress that her father-in-law could not eat properly for several months. While going through a hard time, Ms. Yim’s father-in-law asserted that she should not have included her parents-in-law on the contact list. He vowed to disown Ms. Yim and her husband. It took a long time for Ms. Yim and her husband to convince Ms. Yim’s father-in-law that cooperation with the epidemiological investigation was inevitable.

Ms. Jee (#040), a woman in her forties who was infected at her workplace, recalled a scene from her discharge day. Coming back home, she passed by a supermarket across from her house, and some people who were hanging out there literally ran away as soon as they saw her. “Word must have spread throughout the neighborhood. They looked at me and ran away. The adults took their children’s hands. I was really shocked.”

This outright avoidance of her close neighbors shocked Ms. Jee so deeply that, for a while, she avoided any encounter. She developed a new habit of looking around when she left home to make sure no one was around her. Although she had regularly frequented the market before her COVID-19 infection, she stopped going post-discharge because she believed that she would no longer be welcome. Ms. Jee remarked, “They treated me as if I made a huge mistake. You would not do that, even for sex offenders. They are not strangers; they are my next-door neighbors.”

Due to the government’s policy of posting notices of infected individuals’ routes, some participants were involuntarily detected as infected. Ms. Ahn (#029), who was infected in a club, said, “The epidemiological investigators told me that they would not reveal the name of my store unless I wanted to do so, but the address and name of the store were posted on the city homepage. I got calls from everyone I knew. I was so freaked out and made up a story that a patient had come by my store. I felt like I was being bullied.”

Even when infected individuals’ personal information were not revealed, their neighbors tried to investigate and identify who the infected were out of fear. Although these investigations might be an adaptive behavior to make sure their own safety (to avoid the place, to determine whether or not to get a test, etc.), but, resultingly, these investigations in many cases ended up blames the survivors for their carelessness and made the participants feel guilty and isolation. South Korea has well-established online communities for each local society, and these web-based forums acted as channels through which public opinions and information regarding COVID-19 were shared. Ms. Hahm (#058), who lived in an apartment complex, experienced the following after her family’s infection: “(On the online community of the apartment complex), (t)he neighbors tried to find out which house was the one. It was an atmosphere in which people would say, “Why on earth did they (not stay at home but) thoughtlessly wander around during this pandemic crisis?” People living in the same building posted on the forum, saying, “I have just called my boss, and she told me to take the day off tomorrow because the infected lives in the same building as me. Should I get a test now? Please tell me which floor the infected lives on.” I thought it was just a matter of time before people detected us. I thought people would hate us because they suffered damage because of us. (B)ut I wondered if I really made such a big mistake. I could not stop self-flagellating.”

It is natural for people to expect warm support and empathy from their relatives, friends, and neighbors, especially during a crisis. On the one hand, close relationships were the main source of emotional and financial support for the participants. On the other hand, these close people’s casting of blame and attempts to detect who was infected deeply impacted the participants psychologically. In particular, the negative reactions from neighbors broke their sense of serenity in their homes, and some participants reported that they had considered moving after being infected. When faced with the responses of their relatives, friends, and neighbors, some participants felt skepticism about the social relationships they had built, which caused psychological shrinkage, feelings of isolation, alienation, and depression to the participants.

#### 3.3.3. Contexts in workplaces

Unlike the private networks, the participants hardly controlled the scope and timing of disclosure at their workplaces. Once they reported their infection, this news was immediately delivered to their seniors and teams, and, in nine cases, the whole workforce was notified. For instance, Mr. Eom (#031), who was a soldier, found out that his name as the infected was disclosed to the whole military. Within the military intranet, anyone could figure out his private information, including his university, military service number, and contact number. He said: “It was just like how laypeople know a celebrity, but the celebrity does not know the laypeople. “He is the infected.” People at work knew everything about me, and not because of a good event but because of my COVID-19 infection. This made me scared.”

The sense of causing trouble amplified in terms of workplaces because the participants could not go to the office at least 10 days, and further, their infection resulted in the colleague’s COVID-19 test and quarantine. Particularly, in the case of workplaces, there were many cases that the first patient of serial infection was clear. After returning to work, some participants sensed reinforced policies, such as wearing only KF94 masks, not being allowed to talk during breaks, and undergoing surveillance through CCTV, which made the participants feel guilty.

The perceived and internalized stigma at workplaces caused the participants to feeling of guilt, being overt blamed, rumors, and being distracted. In some cases, involuntary disclosure hampered the participants’ work. After Mr. Oh’s (#032) infection, the epidemiological investigators contacted every business he worked with, and a rumor that Mr. Oh was infected at a political rally, which was regarded as an inappropriate site to visit “during this crisis,” spread among his business contacts. Some of them were so upset that they considered suing Mr. Oh, which intimidated him for a while.

The most distinguishable and critical feature of workplace stigmatization was that survivors could end up experiencing serious economic damage including job loss if they were held responsible for the “trouble” they caused their workplaces. Some participants, such as Mr. Shin (#028), a financial company employee who was hospitalized in an ICU for 3 weeks, reported that he was more worried about whether he would be paid properly and whether he would be dismissed due to the labor loss and great trouble he caused than the actual physical pain. Although Mr. Shin was not face with a threat of layoff, some participants’ concerns turned out to be valid. Ms. Jung (#008) recalled her return: “All hell broke loose at my workplace. About 30 people worked together, and everything stopped for almost 2 weeks. I was totally on my boss’ blocklist because three more people were infected by me. My boss was not willing to let me work again, which caused me a lot of distress.”

Some participants ended up quitting their jobs after returning to their workplaces. Mr. Jin (#041) was working as a facility manager at a middle school when he and his three children were infected by his wife. After the news of his infection broke out, many teachers and students at his school were tested and isolated for 2 weeks. Mr. Jin was very hurt and intimidated when one of the teachers reproached him, “If your wife was so sick, you should have gotten her tested. Why did you do nothing and cause everyone such trouble?” After being released from the 2 weeks isolation, Mr. Jin was faced with an impossible demand from the school: provide a certificate of COVID-19 negative result. In response to this unfair demand, the public health center did nothing except say, “You talk to the school.” Eventually, Mr. Jin quit his job.

A total of 15 participants reported cases similar to Mr. Jin’s. After returning to the workplace, they were asked to quit for different reasons. Some of the participants thought that they deserved it because of the “great damage” they caused to their workplace. Ms. Yim (#036) also felt pressure from her boss and finally submitted her resignation. Recalling her last week at work, she said, “I could not help but cry. I thought, “This company fired me, so why am I working here for them?” I could have quit immediately, but I insisted on working until the end of the month so that I could earn 100,000 won. But I was upset about that.”

#### 3.3.4. Children’s schools

Children’s school was one of the places where the perceived stigma of survivors was most salient. The participants with young children were most worried and anxious about their children’s social lives after infection. In this study, there were no cases of children being exposed to enacted stigma, but the participants were widely concerned about whether their children would be disadvantaged at school or bullied by friends, particularly due to the sense of causing trouble. Since schools did not announce who was infected, the participants could exert control over disclosure in some sense, but the fact that children were involved in it often made handling disclosure more complicated.

At schools and private educational institutes, students’ infections were followed by all their close contacts’ being tested and quarantined for 2 weeks. This caused the participants the greatest psychological pressure and anxiety about bullying. Ms. Pyo (#057) said: “There was an long line at the screening station when one infection case was confirmed at the school. Working mothers had to take the day off to take their children to the screening center and wait there for a couple of hours. I knew what it was like, and it was a great psychological burden when we were infected.” This guilty feeling was observed in participants who were mothers of young children. They were afraid if their children would be teased, blamed, or excluded for causing the troubles to friends and their families. For this reason, many participants with minor children reported that protecting children from COVID-19-related stigma by hiding their infection was the most crucial mission to them.

Officially, schools did not reveal who was infected to protect the children. However, the participants had to juggle with crisis of involuntary disclosure that their child’s infection would become known to other students, particularly through online mom forums and social network services. For instance, Ms. Pyo (#057) related to the group chat for her child’s class. Each class has its own group chat, where the class teacher and all the students’ parents gather. This group chat is supposed to circulate relevant information in real-time, but during the COVID-19 pandemic, it could function as a channel through which information about who was infected was circulated. After Ms. Pyo’s daughter’s infection, the whole class was tested: “Mothers began to report the test results in the group chat, saying, “My kid tested negative.” I could not say anything. One mother said, “It will be so obvious soon who is the (infected) one.” The teacher interrupted and notified the group, “Please report it (the children’s test results) to me directly, not in this group chat.” After this notification, the reports of negative results stopped, and I avoided the awkward situation.”

Regarding the issue of disclosure, some participants addressed the difficulty of having their young children hide their infection. They felt that they had no choice but to hide it and told their children not to talk about the infection as well. However, this instruction confused the children. Every time the participants chose to make up a story of “self-quarantine at home” (not isolation of infected patient) in front of their children, they were worried if the young children knew that their parents were lying.

As seen in Ms. Pyo’s case, it was because of individual teachers’ efforts to protect infected students from involuntary disclosure that no participants reported their children’s exposure to stigmatization. Ms. Joo (#039) and her 9 years-old girl, Ajin, were infected. Ms. Joo thought that they were fortunate because her daughter tested positive during the at-home quarantine period, which was not followed by other students getting tested. Yet, it was natural for the children in Ajin’s class to wonder why Ajin did not come to school. Ajin’s teacher told them that Ajin was absent because of her atopy treatment. Ms. Joo considered this “gloss over” and a “white lie,” to convince the children of the situation from their level. She felt much gratitude toward the teacher for her efforts to protect Ajin from being exposed to social blame as well as her daily calls to Ajin to tell her how the school day was.

#### 3.3.5. Sexual minorities in mass transmissions

In South Korea, mass infections occurred in some LGBTQ clubs in May 2020, and the mass media heavily covered these cases with sensational expressions, such as “94 infected out of gay clubs” (37). In this study, two participants, Mr. Chae (#043) and Mr. Hahn (#046), who identified themselves as sexual minorities who were infected in this mass transmission. It was these two participants who went through most harsh enacted stigma, breach of privacy, and its consequences among the whole participants.

First, while the KDCA did not address concerns regarding hate speech toward sexual minorities and the risk of outing them, LGBTQ patients were directly exposed to the public’s accusations. Combined with the idea that they were infected in “clubs,” which were regarded as inappropriate places to visit during the pandemic, they were regarded to be responsible for both their own infection and troubles to the whole country. Their sexual identities stimulated further blame with the use of homophobic slurs. Reading the newspapers and the replies the papers received that insulted the gay COVID_19 patients from the mass infections, Mr. Chae and Mr. Hahn experienced a traumatic shock to the extent that Mr. Hahn thought of suicide. He said, “I should not have read them, but I had to. Since then, I have felt the urge to jump out of the window during nights of broken sleep. I was prescribed psychotropic medication because of it.”

Although both Mr. Chae and Mr. Hahn voluntarily visited a screening center to get tested even before the KDCA’s recommendation, it did not help lessen the responsibility of transmission or the public blame. They could not receive emotional support from the gay community either. Mr. Chae said, “Many of my friends regarded going to a club as my fault, and I could not confidently claim that I did not do anything wrong.” Both had many relationships severed. Mr. Chae was rejected by someone he was dating after telling that person about his infection. Mr. Hahn recalled, “I had lost half of my friends in the gay community. I drifted apart from them.”

With regard to the exposure of personal affairs, Mr. Hahn was more acutely afflicted than anyone in this study. In the period of his infection, the KDCA revealed the names of infected parties’ worksites, the names of apartments, ages, and whereabouts by time. Being covered by the mass media, Mr. Hahn’s information was publicly exposed. Even the mayor of the city posted his information on the official social network service. When combined, these pieces of information could easily identify Mr. Hahn such that acquaintances were able to determine that the infected person in question was Mr. Hahn. Being continuously asked, “Is this you?” he felt terrified and resentful: “It was more painful and traumatic than getting COVID-19. I was skeptical if I could go back home after discharge and was thinking of moving away.”

Involuntary disclosure posed an additional risk to Mr. Chae and Mr. Hahn, which was the risk of being outed. Inevitably, Mr. Chae had to hide his infection from his parents who were unaware of his sexuality. Mr. Hahn also recounted: “The issue of sexual identity was the hardest thing because even my family, work colleagues, and friends from high school came to know it (my sexuality). Even though I told my mom a good excuse, (she) heard an insult directed at me in her workplace.”

Mr. Chae and Mr. Hahn both reported discrimination from healthcare workers, which was rarely mentioned by the rest of the participants. In Mr. Chae’s case, a staff member from a local healthcare center came to his home to pick him up and instructed him not to use the elevator. As a result, Mr. Chae had to climb down the stairs to the ground floor from his 18th-floor-apartment. The staff also instructed him to wear a protective suit and to spray disinfectant around himself on his way down to the ground. Of the participants, Mr. Chae was the only one who was instructed to do so. Although Mr. Chae was deeply ashamed of this instruction, feeling as though he had become “a carrier,” he thought to himself, “The virus is coming out of me, so I must do this.”

Neither Mr. Chae nor Mr. Hahn could return to work, even after recovery. Mr. Hahn reported that he still had not been able to visit near his workplace at the time of the interview (1 year after his infection). “I could not go back to my work. They said I could, but honestly, everyone knows it (the infected gay) was me. How can I go there? I still cannot go the area, even though it is so close to my home. It would be correct to say that I gave up on being reinstated. The company said I could, but they felt uncomfortable, and I heard a rumor that I would be sent to another branch. I just quit.”

## 4. Discussion

This study showed that the attributes, contexts, consequences, and disclosure management of COVID-19-rerlated stigma that the survivors went through in South Korea in the first 2 years. The main attributes of stigma that were attached to the COVID-19 survivors were the sense of causing trouble and the fear of transmission. The survivors had to cope with both perceived and enacted stigma in their intimate social relationships, workplaces, and children’s schools, ranging from subtle actions to job loss, and some of them reported internalized stigma. While the survivors could control over the disclosure of infection in their private network to some degree, they experienced involuntary disclosure at workplace. Due to the sense of causing trouble and the fear of transmission after the recovery, the survivors could not be treated merely as a patient who needed care and support.

The results of the study revealed that labeling, attributes, and disclosure of COVID-19-related stigma were closely linked not only to the biological characteristics of the disease but also to socio-cultural aspects of the public health preventive measures (3, 38–41). At the core of stigma are difference and deviance (5). The process of taking a screening test, receiving a positive result, being taken away to a healthcare center *via* ambulance, being assigned a case number, having their route posted publicly, and enduring isolation and quarantine maximized the participants’ sense of being distinguished and becoming “other.” The no-test discharge policy played a barrier to the survivor’s return to workplaces and schools, and the public post regarding a patient’s whereabout policy was in part abused to detect who was infected.

Particularly, it is noteworth that the sense of causing trouble appeared as a main attribute. In the participants’ account, “trouble” refers to implicating family, friends, neighbors, and colleagues at work in a test, quarantine at home, and isolation. Through the preventative measure process, the survivors were changed from “a patient” to “a transmitter,” which was susceptible to blaming. The survivors were easily internalized the subdivision those who do not follow public health guidelines (“moral other”) and those who do (“moral us”), viewing the latter as responsible citizens (42), which is in line with the prominent pandemic discourse in Canada (31) and Italy (43). To minimize this accusation, the survivors, on the one hand, utilized the sense of division between innocent victims and those being held responsible for infection transmission while internalizing this blame and trying to see themselves as the former, which reproduced the stigmatization. However, the survivors in this study highlighted that they ended up being blamed regardless of how sincerely they observed the rules and whether they caused any additional infections. To sum up the above, it seems apparent that the preventative measures to some extent played as a role of predisposing and precipitating factors of stigma (6) in South Korea.

The underlying idea of responsibility and causing trouble reflects the belief that the prevention of infectious disease depends upon an individual’s efforts and self-discipline. This silent pressure, both from inside and outside, may function to encourage individuals to discipline themselves (28). However, given that there are no perfect preventive measures against a respiratory infectious disease such as COVID-19, this idea contributed to the survivors’ anxiety, feeling of guilt, and shame.

Another aspect essential to stigma is the fear of transmissibility. This fear has been regarded as the representative driving factor of COVID-19-related stigma (3, 44, 45). Yet, this study discovered the specific context in which the scientific explanation of the remaining virus in the body and the discharge process without a final test together resulted in the fear of post-recovery-transmissibility and barriers to returning to everyday life. This result suggests that how to explain the scientific fact to the public and the processual aspect of public health may be as important as the provision of accurate information (46) in order to mitigate infectious disease-related stigma.

The LGBTQ survivors’ cases in this study revealed the power structure of stigma (47). The high level of stigmatization that these survivors endured is by no means irrelevant to the preexisting stereotypes attached to sexual minorities (e.g., that they carelessly have sex) and the hate discourse against them that has its foundations firmly rooted in the larger social structure (13). This result indicates that socially vulnerable individuals are not only easily targeted as scapegoats (48–50) but also face harsher blame and less social support after infection, regardless of how responsibly they manage their condition (51, 52). Particularly, this study revealed that when a social minority group is engaged in the mass transmission of disease, the public accusation occurs more relentlessly.

## 5. Suggestions

Drawing upon the results of this study, we would like to make some suggestions for the next pandemic. Since stigma is ultimately a matter of power structures, eliminating disease-related stigma must not be left to individuals. Active governmental intervention is needed to reduce the stigma related to COVID-19 and new EIDs (11). The findings of this study indicate the necessity of two layers of efforts: (1) to invent preventative measures that do not contribute creating stigma and (2) to take care of survivors. First, the preventive measures must include how to return the patients to everyday life. The process of diagnosis and isolation plays a significant role in differentiation and otherizing. If so, the government and public health authority are responsible for bringing the patient back to workplace and schools. As reported in the U.S. as well (53), an official certification that those who have finished treatment and been released from isolation/quarantine are no longer transmittable and can return to daily activities can lessen stigmatization. Also, as pointed out in a previous study (6, 54, 55), the provision of accurate and focused information about COVID-19 from a reliable source can reduce stigmatization. Yet, this information should contain not only guidelines to avoid infection and the statistics of infection cases but also guidance on how to support patients and survivors, what survivors should do after recovery, and the meta-message that the infected are not perpetrators but merely patients who need treatment and support.

Third, the government and the mass media industry should reach an agreement on how to cover EIDs and their patients/survivors. As noted, the discriminatory and exclusive metaphors used by some media and newspaper outlets often fuel stigma (11, 42, 55–58) and cause psychological distress to the infected (59). In this study, the stories of “gay club mass infections” left LGBTQ survivors exposed to hate speech and contributed to justifying discrimination against them. Also, as this study reveals, highlighting the collateral damage and inconvenience of individual infection (e.g., how many people had to get a test due to the confirmed case) that resultingly condemns the patients would exacerbate the infectious-disease-related stigma. Further, attention should be given to the social network services and online forums through which public blame, stereotyping, labeling, detecting, and involuntary disclosing are carried out in order to mitigate stigmatization, not only in South Korea but also in other countries (24, 32, 60).

With regard to taking care of the survivors, mental health care for survivors must be provided. Previous studies have reported that COVID-19 patients and survivors may suffer from psychological symptoms, such as post-traumatic stress symptoms, depressive symptoms, insomnia, and suicidal thoughts (61–64). In this study, survivors struggled due to the double bind of stigma: on the one hand, they faced post-recovery discrimination; on the other hand, the fear of stigmatization prevented them from talking about this discrimination and social stigma, as well as the psychological distress that originated from quarantine and the physical isolation they experienced during treatment, which can harm body–mind wellbeing (65, 66). Most of all, more proactive and preemptive approaches to avoid stigma should be taken rather than to counteract already existing patterns of stigma.

## 6. Conclusion

To successfully prevent a new EID, civil cooperation with the policies is crucial, especially, the cooperation of the affected people. The COVID-19-related stigma that survivors have undergone is detrimental to halting and controlling pandemics. If the public health guidelines focus on only the narrow biomedical aspects, this limits our understanding of how policies should be shaped to offer the most effective and equitable response (3). This study has several implications: it reports the unique features of South Korea in East Asia where the preventive measures have unfolded in different ways from Euro-American countries. Particularly, by listening to the voices of the COVID-19 survivors, this study provides empirical evidence of the local context in which the survivors encountered stigmatization.

## 7. Limitation

The limitation of this study is that this study focused on survivors in the first 2 years of the infection onset. Since then, public anxiety and the intensity of preventive measures have decreased. Because stigma is essentially historical and circumstantial (13), further studies are needed to identify the ways in which these changes impact the transformation of stigmatization patterns in South Korea.

## Data availability statement

The datasets presented in this article are not readily available because of participant privacy. Requests to access the datasets should be directed to HYoP, psychepark@gmail.com.

## Ethics statement

The studies involving human participants were reviewed and approved by the Ethics Committees of Seoul National University Hospital (No. H-2010-048-1163), Seoul National University Bundang Hospital, National Medical Center, Armed Forces Capital Hospital, Seongnam Citizens Medical Center, and Boramae Medical Center. Written informed consent for participation was not required for this study in accordance with the national legislation and the institutional requirements.

## Author contributions

HYoP and J-WP: conceptualization. JK, HK, HJY, and YL: data curation. JK, HK, HJY, YL, and HYoP: formal analysis. JK, K-HS, HO, MS, SL, J-WP, and HYoP: methodology. JK: draft of the manuscript. All authors: investigation, critical revision and editing of the manuscript, and contributed to the article and approved the submitted version.
